# Genetic Screening of New Genes Responsible for Cellular Adaptation to Hypoxia Using a Genome-Wide shRNA Library

**DOI:** 10.1371/journal.pone.0035590

**Published:** 2012-04-16

**Authors:** Seiko Yoshino, Toshiro Hara, Jane S. Weng, Yuka Takahashi, Motoharu Seiki, Takeharu Sakamoto

**Affiliations:** Division of Cancer Cell Research, Institute of Medical Science, the University of Tokyo, Minato-ku, Tokyo, Japan; University of Texas Health Science Center at San Antonio, United States of America

## Abstract

Oxygen is a vital requirement for multi-cellular organisms to generate energy and cells have developed multiple compensatory mechanisms to adapt to stressful hypoxic conditions. Such adaptive mechanisms are intricately interconnected with other signaling pathways that regulate cellular functions such as cell growth. However, our understanding of the overall system governing the cellular response to the availability of oxygen remains limited. To identify new genes involved in the response to hypoxic stress, we have performed a genome-wide gene knockdown analysis in human lung carcinoma PC8 cells using an shRNA library carried by a lentiviral vector. The knockdown analysis was performed under both normoxic and hypoxic conditions to identify shRNA sequences enriched or lost in the resulting selected cell populations. Consequently, we identified 56 candidate genes that might contribute to the cellular response to hypoxia. Subsequent individual knockdown of each gene demonstrated that 13 of these have a significant effect upon oxygen-sensitive cell growth. The identification of *BCL2L1*, which encodes a Bcl-2 family protein that plays a role in cell survival by preventing apoptosis, validates the successful design of our screen. The other selected genes have not previously been directly implicated in the cellular response to hypoxia. Interestingly, hypoxia did not directly enhance the expression of any of the identified genes, suggesting that we have identified a new class of genes that have been missed by conventional gene expression analyses to identify hypoxia response genes. Thus, our genetic screening method using a genome-wide shRNA library and the newly-identified genes represent useful tools to analyze the cellular systems that respond to hypoxic stress.

## Introduction

Most eukaryotic cells make use of oxidative phosphorylation (OXPHOS) in the mitochondria for the production of ATP during normoxia. When the oxygen supply becomes insufficient, OXPHOS becomes inefficient and the cell makes use of glycolysis to compensate for this insufficiency. Hypoxia is common in tumor tissues in which the glycolytic activity is usually markedly increased relative to the surrounding non-tumor tissue. This phenomenon can be exploited as a means of early detection of small cancerous nodules by marking the latter specifically with ^18^F-fluorodeoxy glucose, followed by detection of the labeled cells in patients using positron emission tomography (PET) [1], [2]. The transcription factor Hypoxia Inducible Factor-1 (HIF-1) plays a major role in switching ATP production from OXPHOS to glycolysis under hypoxic conditions. In most cells, HIF-1 is inhibited by oxygen sensor proteins, such as HIF prolyl-hydroxylases (PHDs) and Factor Inhibiting HIF-1 (FIH-1) under oxygen-rich conditions [3]–[5]. On the other hand, malignant tumor cells are also known to make use of glycolysis for energy production even during normoxia and this phenomenon is known as the Warburg Effect [6]. Although many genes, such as p53, mTOR, Ras, and Myc, have been implicated in the development of the Warburg effect, HIF-1 is the major player [5], [7], [8].We have studied MT1-MMP, a membrane type matrix metalloprotease (MMP), that promotes cancer cell invasion and growth in a collagen-rich tissue environment via its proteolytic activity. During the course of that study, we also observed that the cytoplasmic tail of MT1-MMP stimulates HIF-1 activity independently from the protease activity and thereby contributes to the Warburg Effect [8]–[10]. It was surprising to us that this membrane protease had an unexpected new function to activate HIF-1. Based on the discovery of the new link between HIF-1 and MT1-MMP, we became interested in the molecular links between different cellular regulatory mechanisms that control the response of cells to hypoxia. Cross talk between these signaling pathways is presumably important for cells to adapt to hypoxic stress in a manner that is coordinated with the regulation of other cellular processes. Activation or inhibition of such connections in cancer cells may benefit cell survival and proliferation.

Some cancer cells survive and even grow under hypoxic conditions that would lead to apoptosis of normal cells. Cancer cells have acquired resistance to apoptosis through the expression of anti-apoptotic proteins such as Bcl-2 and Bcl-xL [11]. In addition, enhanced signaling through Ras, Akt, and mTOR also contributes to the survival and growth of cancer cells during hypoxia [12]–[14]. At the same time, cancer cells tend to adopt a power saving growth strategy by suppressing the transcription and translation of unessential genes. Therefore, it is possible that RNA-protein complexes such as stress granules and processing bodies (P-bodies) regulate the translation of transcripts during hypoxia [15]–[17]. Abnormal proteins generated during translation collect in the endoplasmic reticulum under stressful conditions, such as hypoxia and low nutrient availability. Because the accumulation of excessive levels of such unfolded proteins provokes apoptosis, the appropriate translation and folding/sorting of proteins is expected to be required for cell survival and growth during hypoxia [14].

Since diverse cell functions involving many genes are expected to play roles in cell survival and growth during hypoxia, the cellular responses to hypoxia have been studied extensively. Nevertheless, our understanding of the regulation of cellular responses to hypoxic stress is still fragmentary. This may be because most previous studied have focused on genes whose expression is induced during hypoxia [18]–[20]. To identify new genes involved in the cellular response to hypoxia and its affect upon cell growth, we performed a genetic screen using an shRNA library that covers forty thousand genes. We transduced the shRNA sequences into recipient cells with a lentiviral vector and the cells were selected by growing during either hypoxia or normoxia. The relative abundance of individual shRNA sequences in the selected cell populations presumably reflects the contribution of the target genes of the shRNA sequences to cell growth in an oxygen-sensitive manner.

In this study, we identified 56 such genes, 13 of which were indeed confirmed to modulate cell growth in an oxygen-dependent manner. These genes include *BCL2L1*, whose product is known to play a role in cell survival during hypoxia [21], [22]. However, the functions of the other 12 genes have yet to be implicated in the hypoxic response.

## Results

### A genome-wide shRNA-based screen for genes that contribute to the cellular response to hypoxia

**Figure 1:**
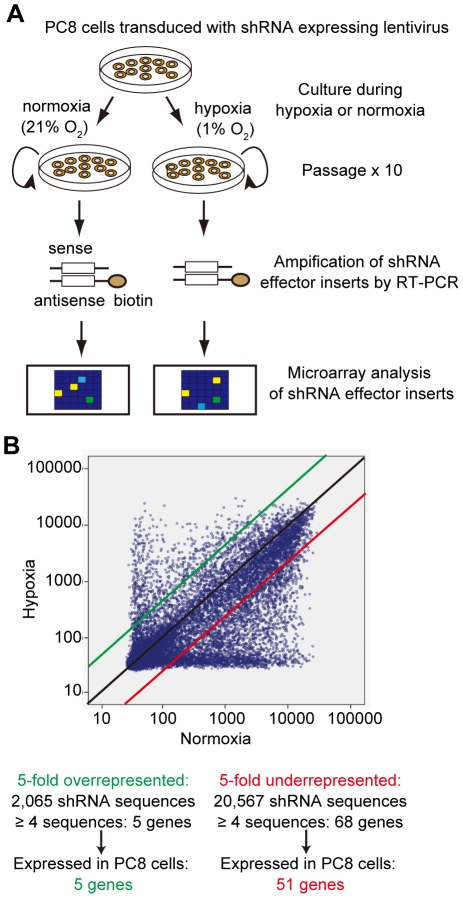
Genome-wide shRNA screen for genes that regulate growth in an oxygen-dependent manner. **A**, Scheme of the genome-wide shRNA screen. Cells transduced with the shRNA library were cultured under normoxia or hypoxia until the tenth passage. The shRNA target sequences were then amplified following reverse transcription PCR (RT-PCR) of total cellular RNA prepared from the selected transfectant pools. The identity and relative abundance of the biotin-labeled shRNA target sequences were evaluated by microarray. **B**, Array analysis of shRNA target sequences amplified from RNA prepared from cells cultured under normoxic or hypoxic selection. Genes targeted by four or more independent shRNA sequences, and which exhibited a representation that differed by more than 5-fold between cells grown under hypoxic and normoxic conditions represent candidate genes that modulate growth in response to the availability of oxygen and were studied further.

We performed a genome wide RNAi-based loss-of-function genetic screen using the Lentivirus GeneNet™ Human 50 K shRNA Library. The library comprises a collection of lentiviral vectors carrying 200,000 short-hairpin RNAs (shRNAs), each targeting one of 38,500 genes. The shRNA sequences were designed to be amplified in vitro so as to allow their analysis by hybridization to Affymetrix GeneChip® Arrays to estimate their relative abundance in the cells. We transduced the library into human lung adenocarcinoma PC8 cells, which were the most permissive among the cancer cell lines tested for transduction by the lentiviral vector. The cells were then cultured under normoxia (21% O_2_) or hypoxia (1% O_2_) until the 10^th^ passage. The shRNA sequences present in the selected cells at that time were then amplified by PCR following reverse transcription (RT-PCR), and the relative abundance of the shRNA sequences was evaluated by microarray analysis (Fig. 1A). The number of shRNA sequences that were more than five times more abundant in cells grown under hypoxia versus normoxia was 2,065 (Fig. 1B). On the other hand, 20,567 shRNA sequences were five times less abundant in the same comparison set (Fig. 1B). Since the library includes four shRNA sequences on average for each gene, we selected genes that were targeted by four or more shRNA sequences. Consequently, 73 genes satisfied this criterion and 56 of these were confirmed to be expressed in PC8 cells by RT-PCR analysis (data not shown). Five genes fell within the “5-fold overrepresented” group and 51 fell within the “5-fold underrepresented” group. A list of these genes is presented in Table S1.

### Effect of the genes in the “5-fold overrepresented” group on cell growth

**Figure 2:**
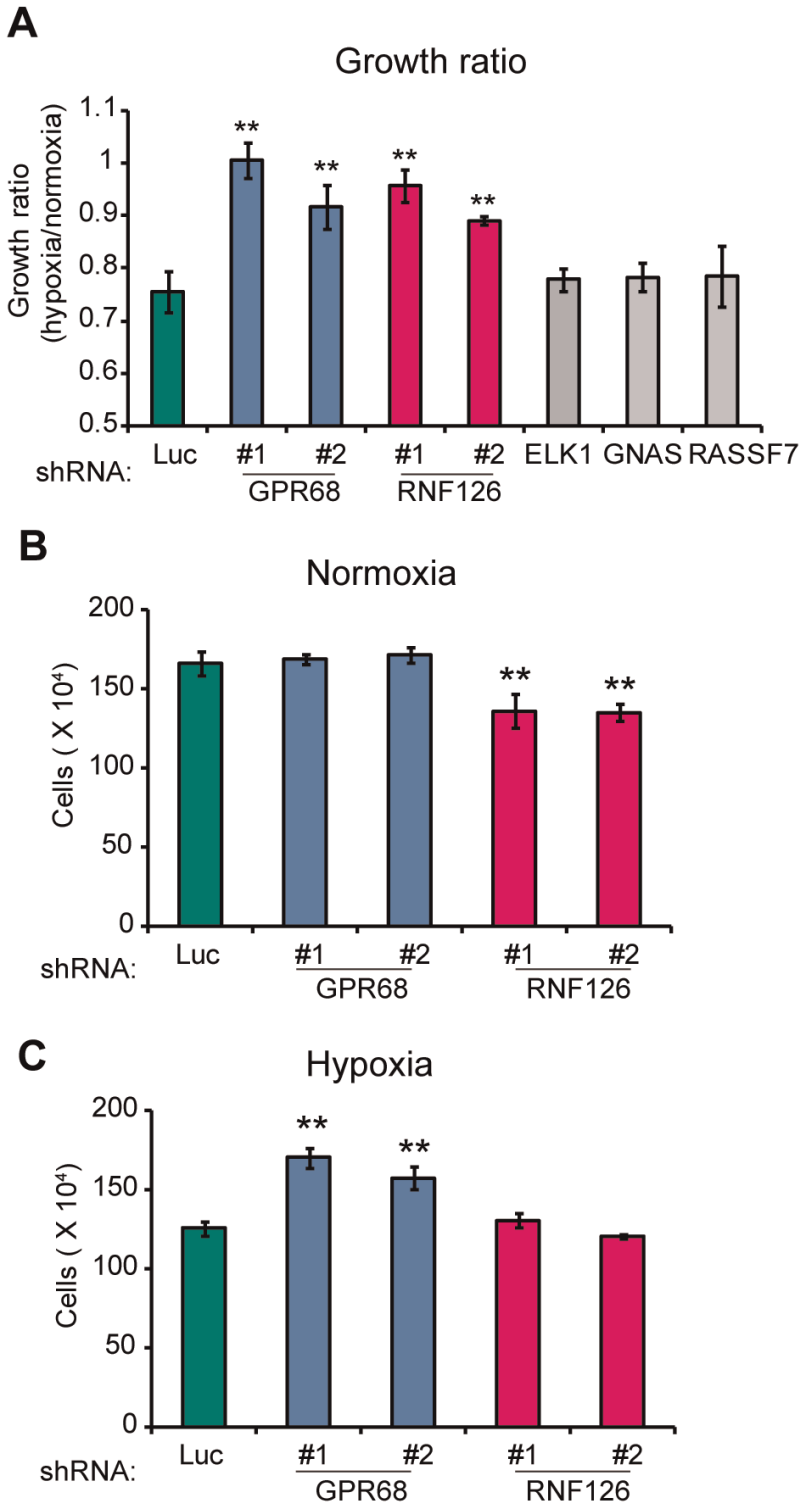
Identification of oxygen-sensitive genes with increased hypoxia/normoxia growth ratio when knocked-down. **A–C**, Confirmatory analysis oxygen-sensitivity of genes indentified in the genome-wide screen. PC8 cells were transduced with lentiviral vectors expressing two independent shRNAs targeting each candidate gene and cultured for one week under normoxia or hypoxia. The resulting hypoxia/normoxia growth ratio (**A**) and cells numbers when cultured during normoxia (**B**) or hypoxia (**C**) were analyzed. In **A–C**, error bars indicate s.d. (n = 3) and the data were analyzed by the t-test. *p<0.05, **p<0.01.

We next knocked down the expression of the 5 genes in the “5-fold overrepresented” group by expressing shRNA sequences targeting each gene using the lentivirus vector system. Knockdown efficiency of each gene was confirmed by RT-PCR analysis to be greater than 70% (data not shown). The transfectants were then cultured under normoxia or hypoxia for seven days. The ratios of cell growth of the transfectants during hypoxia versus normoxia were compared to that of the control cells that expressed an shRNA targeting the luciferase gene (Luc). We observed a significant increase in the ratio of cell growth during hypoxia versus normoxia following stable knockdown of *GPR68* and *RNF126* with either of two different shRNA sequences, but the effect of knockdown of the other three genes was not significant (Fig. 2A). The findings for GPR68 and RNF126 are consistent with the enrichment of the corresponding shRNA sequences during the library screening. Even though the relative growth ratio of the *GPR68* and *RNF126* knockdown cells between hypoxia versus normoxia showed a similar increase, there was a differential effect of the knockdown of each gene on cell growth during hypoxia and normoxia. Specifically, knockdown of *GPR68* increased cell growth during hypoxia but had no effect during normoxia (Fig. 2B, C), and consequently the relative growth ratio between cells grown under hypoxia versus normoxia was increased. In contrast, knockdown of *RNF126* did not affect cell growth during hypoxia but inhibited growth during normoxia (Fig. 2B, C), thus also giving rise to an increase of in the relative growth ratio.

The knockdown of the other three genes had no significant effect upon cell growth, but we do not necessarily believe that this means that these three genes are merely artifacts of the screen. The cell growth analysis over 7 days represents a much shorter period of time than that used for the 10-passage screen, and may be insufficient to observe a measurable effect. Therefore, it is possible that these genes also play roles in the cellular response to hypoxia and that they affect the growth of PC8 cells. In contrast, it is quite clear that *GPR68* and *RNF126* mediate a greater effect on cell growth compared to other three genes.

### Effect of the genes in the “5-fold underrepresented” group

**Figure 3:**
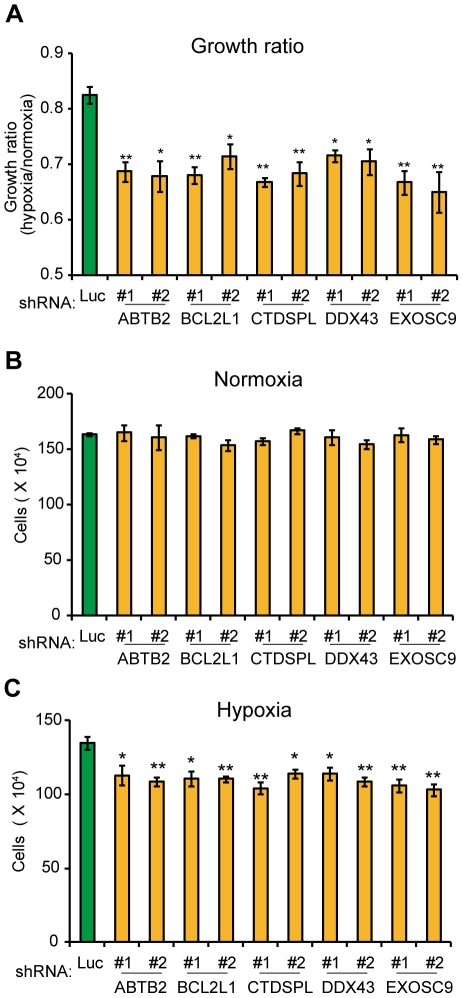
Knockdown of the genes decreased hypoxia/normoxia growth ratio due to reduced growth during hypoxia. **A–C**, Confirmatory analysis of the oxygen-sensitive genes was performed as described in Figure 2. The resulting hypoxia/normoxia growth ratio (**A**) and cell numbers when cultured during normoxia (**B**) or hypoxia (**C**) were analyzed. In **A–C**, error bars indicate s.d. (n = 3) and the data were analyzed by the t-test. *p<0.05.

**Figure 4:**
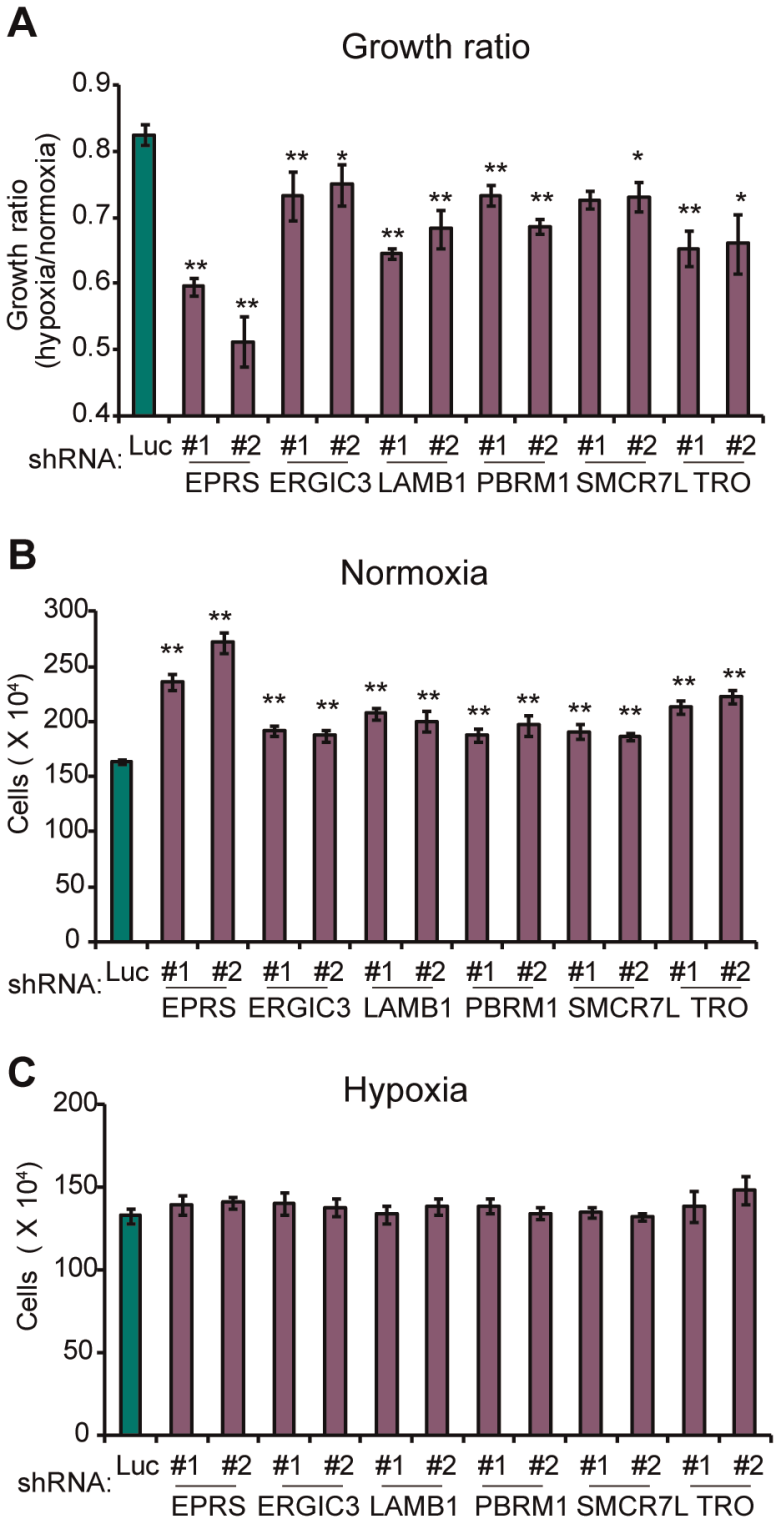
Knockdown of the genes decreased hypoxia/normoxia growth ratio due to increased growth during normoxia. **A–C**, Confirmatory analysis of the oxygen-sensitive genes was performed as described in Figure 2. The resulting hypoxia/normoxia growth ratio (**A**) and cell numbers when cultured during normoxia (**B**) or hypoxia (**C**) were analyzed. In **A–C**, error bars indicate s.d. (n = 3) and the data were analyzed by the t-test. *p<0.05, **p<0.01.

We next carried out knockdown of the 51 genes selected as the “5-fold underrepresented” group in PC8 cells. Among these, knockdown of 11 genes (*BCL2L1*, *DDX43*, *ABTB2*, *EXOSC9*, *CTDSPL*, *LAMB1*, *SMCR7L*, *ERGIC3*, *PBRM1*, *TRO*, *EPRS*) generated cells that exhibited a decreased growth ratio between hypoxia versus normoxia compared to control cells (Fig. 3A and 4A). The growth ratio of the other knockdown cells was comparable to that of the control cells (data not shown). The selected genes could be divided into two groups: One group comprises genes that decreased cell growth during hypoxia but not during normoxia following knockdown (Fig. 3B, C), and the other comprises genes that markedly increased the rate of cell growth during normoxia, but more modestly during hypoxia following knockdown (Fig. 4B, C).

Taken together, the products of the 11 genes in the “5-fold underrepresented” group were confirmed to play roles in the cellular response to hypoxia and to affect cell growth significantly. The effect of other selected gene products on cell growth was negligible at least under the specific conditions of this assay.

### Annotation of the identified genes

We queried the REACTOME (www.reactome.org), PANTHER (www.pantherdb.org), and KEGG databases (www.genome.jp/kegg/) to determine whether any of the 13 identified genes had previously been linked to cellular adaptation to hypoxia or cell growth. Interestingly, *BCL2L1* has an oxygen-sensitive function. Namely, it encodes an anti-apoptotic Bcl-2 family protein Bcl-xL that localizes to the mitochondrial outer membrane and which regulates the opening of an outer membrane channel and as such plays a role in hypoxia-sensitive apoptosis [21], [22]. However, the other 12 genes had not yet been annotated to effect hypoxia related functions.

We next carried out a Gene Ontology analysis of the genes by taking advantage of the AmiGO database (http://amigo.geneontology.org) so as to obtain information about the subcellular localizations of their products. As listed in Table 1, the gene products are likely to localize to the nucleus, cytosol, mitochondria, ER/Golgi, plasma membrane, and extracellular compartments. However, there was no information available for the *CTDSPL* and *RNF126* gene products, so we used data from the PSORT database (psort.hgc.jp) to predict their subcellular localizations. Both products were predicted to localize to the nucleus. Proteins encoded by *GPR68*, *LAMB1*, and *TRO* are likely to localize to the plasma membrane and may face the extracellular space.

**Table 1:** Gene ontology analysis of the identified oxygen-sensitive growth regulator genes.

**Component:**	
Nucleus	BCL2L1, DDX43, EXOSC9, CTDSPL, PBRM1, TRO
Cytosol	BCL2L1, DDX43, EXOSC9, TRO, EPRS
Mitochondrion	BCL2L1, SMCR7L
ER/Golgi	ERGIC3
Plasma membrane	TRO, GPR68
Extracellular region	LAMB1
Unknown	ABTB2, RNF126
**Biological process:**	
Growth/proliferation (both positively or negatively)	ABTB2, BCL2L1, EXOSC9, LAMB1, PBRM1, TRO,
Immune/inflammation response	BCL2L1, EXOSC9, GPR68
Adhesion	LAMB1, TRO
**Molecular function:**	
Protein binding	BCL2L1, EXOSC9, LAMB1, SMCR7L, PBRM1, EPRS, RNF126
RNA binding	DDX43, EXOSC9, EPRS
DNA binding	ABTB2, PBRM1
ATP binding	DDX43, EPRS
Metal ion binding	CTDSPL, RNF126

Categories of biological processes and molecular functions represented by at least two genes are listed.

We next analyzed the annotated biological functions of the identified genes (Table 1, biological process). A role in cell growth and proliferation was annotated to 6 genes, which seems reasonable since the genes were identified based on a screen for oxygen-sensitive cell growth. A role in the immune/inflammation response was annotated to three genes (*BCL2L1*, *EXOSC9*, *GPR68*). As inflammatory sites tend to be hypoxic, these functions of these three genes may be relevant to the control of cell growth during inflammation.

We also explored the annotated molecular functions of the genes (Table 1, molecular function). Protein binding was annotated to six genes and nucleotide binding to either RNA and/or DNA was annotated to five genes, including *DDX43, EXOSC9, EPRS, ABTB2, PBRM1*. Products of these genes may play roles in regulating translation/transcription during the cellular response to stress.

### Identified genes are not inducible during hypoxia

**Figure 5:**
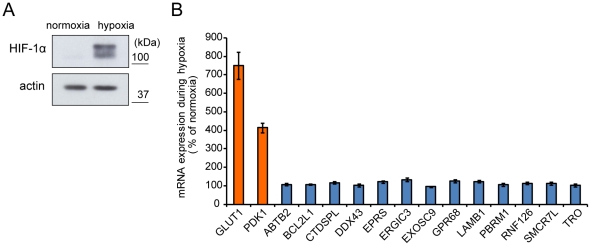
The identified oxygen-sensitive growth regulator genes that are not differentially expressed during hypoxia. **A**, Western blot analysis of actin and HIF-1α in normoxic and hypoxic cells. HIF-1α serves as an indicator of the hypoxic response. **B**, RT-PCR analysis of HIF-1 target genes and the identified oxygen-sensitive growth regulator genes. The expression of each gene was normalized to that of beta-actin. GLUT1 and PDK1 are representatives of HIF-1 target genes.

The increased activity of HIF transcription factors during hypoxia induces the expression of many genes whose products play a role in the adaptation to hypoxia. Indeed, a marked increase in the expression of the HIF-1α protein was observed in PC8 cells cultured for 24 hrs in hypoxic conditions (Fig. 5A). Therefore, it is possible that the expression of the genes selected by the screening is also affected by hypoxia at the transcription level. To evaluate this, we examined the expression of the mRNAs transcribed from the 13 genes during normoxia and hypoxia by quantitative RT-PCR. The mRNAs encoding the representative HIF-1 target genes, *GLUT1* and *PDK1* [23]–[25], were increased approximately by seven and four fold respectively over that observed during normoxia (Fig. 5B). In contrast, none of the 13 identified genes exhibited significantly altered expression during hypoxia (Fig. 5B). Thus, our screening method successfully identified a new class of genes that play roles in the cellular response to hypoxia and which modulate cell growth, but which are not regulated at the level of transcription in a hypoxia-dependent manner.

## Discussion

In this study, we sought to identify genes that play roles in the cellular response to hypoxia. We carried out genome-wide gene knockdown screening using an shRNA library. Target genes that affect cell growth differentially during hypoxia and nomoxia were selected. We identified 73 genes whose mRNAs were targeted by at least four independent shRNA sequences in the library, and whose representation within the selected cell populations exhibited at least a five-fold change between cells grown under normoxia versus hypoxia. Fifty-six of these genes were confirmed to be expressed in PC8 cells, and knockdown of 13 of these genes indeed affected cell growth significantly in an oxygen-dependent manner. Cells depleted for the expression of each identified gene using cognate shRNAs exhibited an approximate 20% difference in the rates of growth when cultured under normoxic versus hypoxic conditions. These effects were significant and reproducible, supporting the screening results. Surprisingly, none of the 13 identified genes is a target of HIF-1 as their expression was not increased during hypoxia (Fig. 5B). However, this result does not necessarily means that HIF-1 and its target genes are not required for the oxygen-sensitive cell growth. Indeed, we detected three or less shRNA sequences for *HIF1A* and HIF-1 target genes such as *GLUT1* and *PDK1* in the list of shRNA screening described in Fig. 1A. Thus, our very stringent selection criteria, namely four or more shRNA sequences for a gene, eliminated HIF-1 and its target genes. This also means that we can identify more genes that potentially regulate the oxygen-sensitive cell growth if stringency of selection criteria was lowered.

The proteins encoded by the 13 genes are possible regulators of the cellular response to hypoxia and consequently affect cell growth. Although these genes have not been implicated in cell growth regulation directly, it will be necessary to identify the reported functions of the gene products before exploring the exact mechanism of the effect of each gene during hypoxia. It is particularly important that the identified genes include *BCL2L1*, which encodes a member of the Bcl-2 protein family and is expected to contribute to cell survival during hypoxia by preventing apoptosis [21], [22]. Therefore, we think the identification of *BCL2L1* validates our screening method and suggests that the conditions applied were appropriate to enrich for the genes we intended. We also speculate that the other gene products may play roles in oxygen-dependent cellular responses that are closely linked to cell growth. *GPR68* encodes a G protein-coupled receptor that binds protons and acts as an extracellular pH sensor [26], [27]. Since hypoxia promotes lactate production via glycolysis, GPR68 may act to sense the acidic extracellular environment so as to transduce a proton-mediated signal to the cell growth machinery. *DDX43*, *EXOSC9*, *CTDSPL*, and *EPRS* genes have been implicated in RNA metabolism [28]–[31]. Because the expression of mRNAs encoding many genes must change to adapt to hypoxia, such as those regulated by HIF transcription factors, the identified genes that encode proteins involved in RNA metabolism might play roles in the transcriptional and/or translational regulation of genes during the adaptation to hypoxia. *CTDSPL* has been implicated as a tumor suppressor gene, and its expression is suppressed or lost in many cancer cell lines [29], [32], [33]. However, *CTDSPL* is expressed in PC8 cells and has a growth-promoting effect during hypoxia (Fig. 3C). As the function of the *CTDSPL* gene product remains unclear, further analyses of *CTDSPL* will be necessary to solve the apparent contradiction between our findings and the reported tumor suppressor function.

In addition to PC8 cells, we examined whether the identified 13 genes also affect the oxygen-sensitive cell growth in another cell line, invasive breast cancer MDA-MB-231 cells (Fig. S1). All genes except *ABTB2* and *DDX43* affected the oxygen-sensitive growth of MAD-MB-231 cells like PC8 cells. *DDX43* was not expressed in MDA-MB-231 cells, whereas *ABTB2* was expressed. Thus, it is plausible that roles of the identified genes contribute to the oxygen-sensitive growth in a cell-context dependent manner.

In summary, we have identified a new class of genes involved in the cellular response to hypoxia using a genome-wide shRNA library-based screen. Identification and analysis of the products of the genes identified by this screen should eventually expand our understanding of the changes in cellular physiology in cells exposed to hypoxic conditions and may provide clues for therapeutic targets to treat diseases where hypoxia plays an important role in the pathophysiology, such as brain and heart infarction, diabetic gangrene, chronic inflammatory disease, and cancer.

## Materials and Methods

### Cell culture

Human lung adenocarcinoma PC8 cells [34] were kindly gifted from National Cancer Center (Tokyo, Japan). Human breast cancer MDA-MB-231 cells were purchased from ATCC. Cells were cultured in RPMI (PC8) or DMEM (MDA-MB-231) containing 10% fetal bovine serum, 100 units/mL penicillin, and 100 µg/mL streptomycin (Sigma) at 37°C in a humidified 5% CO_2_ environment. For experiments conducted under hypoxic conditions, cells were cultured in a 1% O_2_ and 5% CO_2_ environment in a Model 9200 incubator (Wakenyaku).

### RNAi screening

GeneNet™ Lentiviral shRNA Libraries Human 50 K (System Biosciences) were introduced into PC8 cells according to the manufacture's instruction. Library cells were cultured under normoxia or hypoxia until the tenth passage. The shRNA templates were recovered from the selected cells by RT-PCR and analyzed using Affymetrix GeneChip® Arrays according to the manufacture's instruction.

### Vector construction

The shRNA sequences used in this study are listed in Table S2. The deoxyribose versions of the targeted gene sequences were subcloned into pENTR/U6 TOPO (Invitrogen) before being transferred v*i*a recombination into the lentivirus vector, pLenti6 BLOCKi (Invitrogen).

### Cell growth assay

Cells (1×10^4^) were seeded onto a plastic dish and cultured at 37°C in a humidified CO_2_ incubator. The cells were counted periodically using a Coulter counter (Beckman).

### Western blot analysis

Cells were lysed with lysis buffer and centrifuged at 20,000 *g* for 15 minutes at 4°C. The supernatants were collected and total protein content measured using the Bradford assay (Bio-Rad). Lysates were separated by SDS-PAGE, transferred to membrane filters, and analyzed by Western blot using anti-actin mouse antibody (Millipore) or anti-HIF-1α mouse antibody (BD Biosciences).

### RNA isolation, reverse transcription and real-time PCR

Total RNA was isolated from cells using TRIzol reagent (Invitrogen) and subjected to reverse transcription (RT) using SuperscriptII (Invitrogen) and random primers. The RT products were then subjected to real-time PCR in a Smart Cycler II System (Cepheid) using SYBR GREEN I (BioWhittaker Molecular Applications) and the specific primers for each gene (Table S3). The PCR products were sequenced and their homogeneity was confirmed by dissociation temperature monitoring of SYBR GREEN I fluorescence.

### Statistical analysis

We compared two subject groups by the two-sided t-test.

## Supporting Information

Figure S1 **Knockdown of the identified oxygen-sensitive genes affected hypoxia/normoxia growth ratio in MDA-MB-231 cells.** The hypoxia/normoxia growth ratio in MDA-MB-231 cells was analyzed as described in Figure 2. Error bars indicate s.d. (n = 3) and the data were analyzed by the t-test. *p<0.05. N.D.; not detected.
(PDF)

Table S1 **List of “5-fold overrepresented” and “5-fold under represented” groups.**
(PDF)

Table S2 **shRNA sequences used in this study.**
(PDF)

Table S3 **Primer sequences used in this study.**
(PDF)

## References

[1] Cairns RA, Harris IS, Mak TW (2011). Regulation of cancer cell metabolism.. Nat Rev Cancer.

[2] Gatenby RA, Gillies RJ (2004). Why do cancers have high aerobic glycolysis?. Nat Rev Cancer.

[3] Denko NC (2008). Hypoxia, HIF1 and glucose metabolism in the solid tumour.. Nat Rev Cancer.

[4] Kaelin WG, Ratcliffe PJ (2008). Oxygen sensing by metazoans: the central role of the HIF hydroxylase pathway.. Mol Cell.

[5] Semenza GL (2009). HIF-1: upstream and downstream of cancer metabolism.. Curr Opin Genet Dev.

[6] Warburg O (1956). On the origin of cancer cells.. Science.

[7] Luo W, Hu H, Chang R, Zhong J, Knabel M (2011). Pyruvate kinase M2 is a PHD3-stimulated coactivator for hypoxia-inducible factor 1.. Cell.

[8] Sakamoto T, Niiya D, Seiki M (2011). Targeting the Warburg effect that arises in tumor cells expressing membrane type-1 matrix metalloproteinase.. J Biol Chem.

[9] Sakamoto T, Seiki M (2009). Mint3 enhances the activity of hypoxia-inducible factor-1 (HIF-1) in macrophages by suppressing the activity of factor inhibiting HIF-1.. J Biol Chem.

[10] Sakamoto T, Seiki M (2010). A membrane protease regulates energy production in macrophages by activating hypoxia-inducible factor-1 via a non-proteolytic mechanism.. J Biol Chem.

[11] Zhou J, Schmid T, Schnitzer S, Brune B (2006). Tumor hypoxia and cancer progression.. Cancer Lett.

[12] Kroemer G, Pouyssegur J (2008). Tumor cell metabolism: cancer's Achilles' heel.. Cancer Cell.

[13] Manning BD, Cantley LC (2007). AKT/PKB signaling: navigating downstream.. Cell.

[14] Wouters BG, Koritzinsky M (2008). Hypoxia signalling through mTOR and the unfolded protein response in cancer.. Nat Rev Cancer.

[15] Anderson P, Kedersha N (2009). RNA granules: post-transcriptional and epigenetic modulators of gene expression.. Nat Rev Mol Cell Biol.

[16] Parker R, Sheth U (2007). P bodies and the control of mRNA translation and degradation.. Mol Cell.

[17] Spriggs KA, Bushell M, Willis AE (2010). Translational regulation of gene expression during conditions of cell stress.. Mol Cell.

[18] Hu CJ, Wang LY, Chodosh LA, Keith B, Simon MC (2003). Differential roles of hypoxia-inducible factor 1alpha (HIF-1alpha) and HIF-2alpha in hypoxic gene regulation.. Mol Cell Biol.

[19] Kunz M, Moeller S, Koczan D, Lorenz P, Wenger RH (2003). Mechanisms of hypoxic gene regulation of angiogenesis factor Cyr61 in melanoma cells.. J Biol Chem.

[20] Zhang L, Hill RP (2004). Hypoxia enhances metastatic efficiency by up-regulating Mdm2 in KHT cells and increasing resistance to apoptosis.. Cancer Res.

[21] Shimizu S, Eguchi Y, Kamiike W, Itoh Y, Hasegawa J (1996). Induction of apoptosis as well as necrosis by hypoxia and predominant prevention of apoptosis by Bcl-2 and Bcl-XL.. Cancer Res.

[22] Shimizu S, Eguchi Y, Kosaka H, Kamiike W, Matsuda H (1995). Prevention of hypoxia-induced cell death by Bcl-2 and Bcl-xL.. Nature.

[23] Huang J, Zhao Q, Mooney SM, Lee FS (2002). Sequence determinants in hypoxia-inducible factor-1alpha for hydroxylation by the prolyl hydroxylases PHD1, PHD2, and PHD3.. J Biol Chem.

[24] Kim JW, Tchernyshyov I, Semenza GL, Dang CV (2006). HIF-1-mediated expression of pyruvate dehydrogenase kinase: a metabolic switch required for cellular adaptation to hypoxia.. Cell Metab.

[25] Papandreou I, Cairns RA, Fontana L, Lim AL, Denko NC (2006). HIF-1 mediates adaptation to hypoxia by actively downregulating mitochondrial oxygen consumption.. Cell Metab.

[26] Huang WC, Swietach P, Vaughan-Jones RD, Ansorge O, Glitsch MD (2008). Extracellular acidification elicits spatially and temporally distinct Ca2+ signals.. Curr Biol.

[27] Ludwig MG, Vanek M, Guerini D, Gasser JA, Jones CE (2003). Proton-sensing G-protein-coupled receptors.. Nature.

[28] Arif A, Jia J, Mukhopadhyay R, Willard B, Kinter M (2009). Two-site phosphorylation of EPRS coordinates multimodal regulation of noncanonical translational control activity.. Mol Cell.

[29] Kashuba VI, Li J, Wang F, Senchenko VN, Protopopov A (2004). RBSP3 (HYA22) is a tumor suppressor gene implicated in major epithelial malignancies.. Proc Natl Acad Sci U S A.

[30] Martelange V, De Smet C, De Plaen E, Lurquin C, Boon T (2000). Identification on a human sarcoma of two new genes with tumor-specific expression.. Cancer Res.

[31] van Dijk EL, Schilders G, Pruijn GJ (2007). Human cell growth requires a functional cytoplasmic exosome, which is involved in various mRNA decay pathways.. Rna.

[32] Ghosh A, Ghosh S, Maiti GP, Sabbir MG, Zabarovsky ER (2010). Frequent alterations of the candidate genes hMLH1, ITGA9 and RBSP3 in early dysplastic lesions of head and neck: clinical and prognostic significance.. Cancer Sci.

[33] Senchenko VN, Anedchenko EA, Kondratieva TT, Krasnov GS, Dmitriev AA (2010). Simultaneous down-regulation of tumor suppressor genes RBSP3/CTDSPL, NPRL2/G21 and RASSF1A in primary non-small cell lung cancer.. BMC Cancer.

[34] Shinmoto H, Dosako S, Yamada K, Shirahata S, Murakami H (1990). Purification and some properties of proliferation suppressing factor from human lung adenocarcinoma PC-8.. Cytotechnology.

