# Supplementary figures and images for: Genetic Screening of New Genes Responsible for Cellular Adaptation to Hypoxia Using a Genome-Wide shRNA Library
Source: PLoS One. 2012 Apr 16;7(4):e35590. doi: 10.1371/journal.pone.0035590 (PMC3327663; doi:10.1371/journal.pone.0035590)

Figure S1

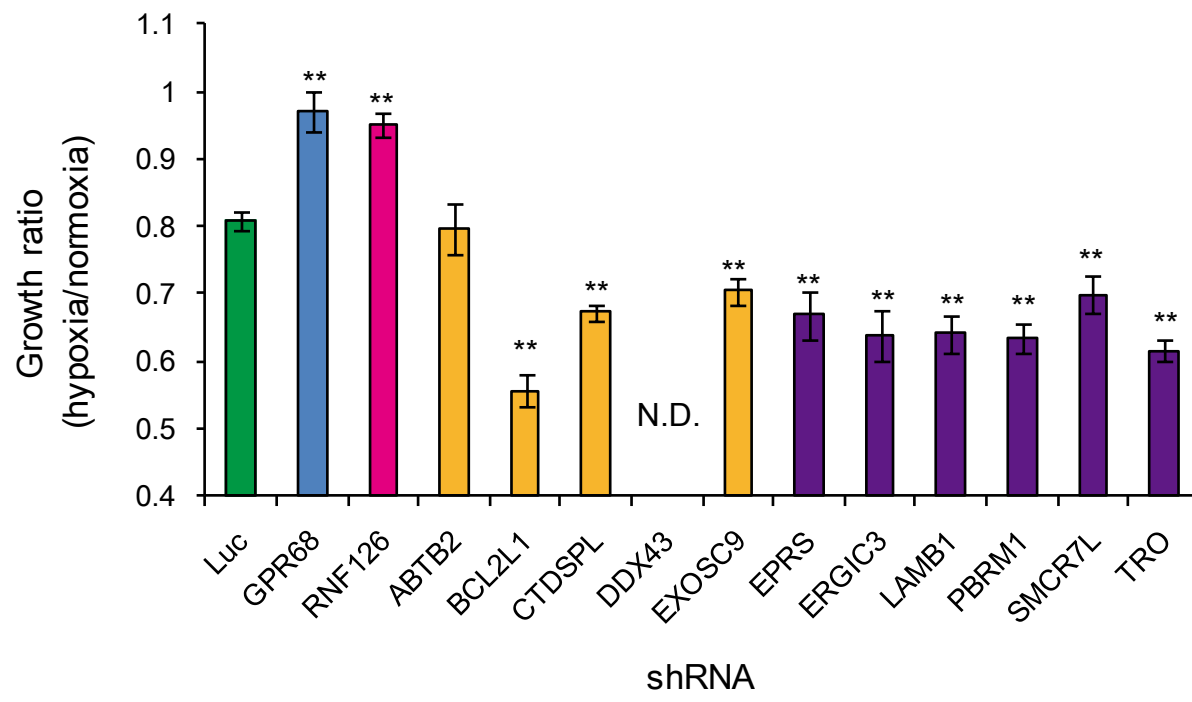

Supplement: Figure S1 — Knockdown of the identified oxygen-sensitive genes affected hypoxia/normoxia growth ratio in MDA-MB-231 cells. The hypoxia/normoxia growth ratio in MDA-MB-231 cells was analyzed as described in Figure 2. Error bars indicate s.d. (n = 3) and the data were analyzed by the t-test. *p<0.05. N.D.; not detected. (PDF) [file pone.0035590.s001.pdf]
