# Supplementary material for: Genetic Screening of New Genes Responsible for Cellular Adaptation to Hypoxia Using a Genome-Wide shRNA Library
Source: PLoS One. 2012 Apr 16;7(4):e35590. doi: 10.1371/journal.pone.0035590 (PMC3327663; doi:10.1371/journal.pone.0035590)
Supplement: Table S1 — List of “5-fold overrepresented” and “5-fold under represented” groups. (PDF) [file pone.0035590.s002.pdf]

Table S1

List of “5-fold overrepresented” and “5-fold under represented” groups.

“5-fold overrepresented” group (5 genes)
ELK1, GNAS, GPR68, RASSF7, RNF126
“5-fold underrepresented” group (51 genes)
ABTB2, ACAT2, ANPEP, ARFGEF1, BCL2L1, C11orf80, CANX, CCDC9, CCDC19, CCPG1, CENTG1, CGB, COIL, CREM, CROT, CTDSPL, DAPK2, DDX43, DNM1L, EDG2, EPRS, ERGIC3, EXOSC9, FLI1, GOSR1, HEXIM1, HEYL, KLRC3, LAMB1, LAX1, LXN, MFSD10, MGC4172, NFYC, NIP30, NRG1, PBRM1, PCGF2, PHF8, PHF20, PLCE, PSMF1, SDAD1, SLC35D1, SMCR7L, SPIN2B, ST3GAL3, TGFB1, TMEM41B, TNIK, TRO
