# Supplementary material for: Genetic Screening of New Genes Responsible for Cellular Adaptation to Hypoxia Using a Genome-Wide shRNA Library
Source: PLoS One. 2012 Apr 16;7(4):e35590. doi: 10.1371/journal.pone.0035590 (PMC3327663; doi:10.1371/journal.pone.0035590)
Supplement: Table S2 — shRNA sequences used in this study. (PDF) [file pone.0035590.s003.pdf]

Table S2

Luc (control)		GCAUCACGUACGCGGAAUACCGAAGUAUUCCGCGUACGUGAUG
overrepresented:		
GPR68	#1	GGAACAUCACUGCAGACAACUCGAAAGUUGUCUGCAGUGAUGUUCC
	#2	GCUGUGUCUCUUAGCCCUUUCCGAAGAAAGGGCUAAGAGACACAGC
RNF126	#1	GCUUUGAAAUAAACGGACGUUCGAAAACGUCCGUUUAUUUCAAAGC
	#2	GCACUCAAACCCUAUGGACUACGAAUAGUCCAUAGGGUUUGAGUGC
underrepresented:		
BCL2L1	#1	GCUCUCUGCUGUACAUAUUUGCGAACAAAUAUGUACAGCAGAGAGC
	#2	GCAAUUCCUGUGUCGCCUUCUCGAAAGAAGGCGACACAGGAAUUGC
DDX43	#1	GCCAUUGAUAGAUUGGGAUCACGAAUGAUCCCAAUCUAUCAAUGGC
	#2	GCAAGGAAUAGAUCUUAUAGGCGAACCUAUAAGAUCUAUUCCUUGC
ABTB2	#1	GCGUGCACUCUGUCUACAUCACGAAUGAUGUAGACAGAGUGCACGC
	#2	GGCUCCUUUAAUGUAGGAUUGCGAACAAUCCUACAUUAAAGGAGCC
EXOSC9	#1	GCAAAUACGUGUAGACCUACACGAAUGUAGGUCUACACGUAUUUGC
	#2	GCGUGAUCCUGUACCAUUAAGCGAACUUAAUGGUACAGGAUCACGC
CTDSPL	#1	GCUCCUGAUAGGAGGAUUUCACGAAUGAAAUCCUCCUAUCAGGAGC
	#2	GCUUGACUAUGGAAAGAAAUGCGAACAUUUCUUUCCAUAGUCAAGC
LAMB1	#1	GCAAUUCCCAAGAUCCUUAUCCGAAGAUAAGGAUCUUGGGAAUUGC
	#2	GGUUGUAAAUCUUGUGCUUGCCGAAGCAAGCACAAGAUUUACAACC
SMCR7L	#1	GCCACUGAAGAGCUGUGAUAGCGAACUAUCACAGCUCUUCAGUGGC
	#2	GCACCACUUCAUCAUGAAUGCCGAAGCAUUCAUGAUGAAGUGGUGC
ERGIC3	#1	GCUUCCUCUGUCUCCUCUUUCCGAAGAAAGAGGAGACAGAGGAAGC
	#2	GCACAUGCCUUGUGCCUAUCUCGAAAGAUAGGCACAAGGCAUGUGC
PBRM1	#1	GCAUCUGUCUGCAGCUAAUGUCGAAACAUUAGCUGCAGACAGAUGC
	#2	GCUACCGUCGGCUUGAUUUAUCGAAAUAAAUCAAGCCGACGGUAGC
TRO	#1	GGCUAAUGGUAGGAAUGUUAACGAAUUAACAUUCCUACCAUUAGCC
	#2	GGAUGUCAUCCAAGAAUAUGACGAAUCAUAUUCUUGGAUGACAUCC
EPRS	#1	GCAACUACAGCUGGGUUAUAUCGAAAUAUAACCCAGCUGUAGUUGC
	#2	GGAACAUACUGAGAUUGAUCACGAAUGAUCAAUCUCAGUAUGUUCC
