# Supplementary material for: Genetic Screening of New Genes Responsible for Cellular Adaptation to Hypoxia Using a Genome-Wide shRNA Library
Source: PLoS One. 2012 Apr 16;7(4):e35590. doi: 10.1371/journal.pone.0035590 (PMC3327663; doi:10.1371/journal.pone.0035590)
Supplement: Table S3 — Primer sequences used in this study. (PDF) [file pone.0035590.s004.pdf]

Table S3

**Control gene:**		
beta-actin	Sense (S)	5’-GGGACGACATGGAGAAAATC-3’
	Antisense (A)	5’-GGGTGTTGAAGGTCTCAAAC-3’
**HIF-1 target genes:**		
GLUT1	S	GGGCATGTGCTTCCAGTATGT
	A	ACCAGGAGCACAGTGAAGAT
PDK1	S	TCCTGTCACCAGCCAGAATG
	A	CTTCCTTTGCCTTTTCCACC
**Identified genes:**		
ABTB2	S	GTGACCGAGGGCTTGCAGCT
	A	CCAGGAAGGTCACATCTGAC
BLC2L1	S	GGGCATTCAGTGACCTGACA
	A	AACCAGCGGTTGAAGCGTTC
CTDSPL	S	TACGTGAAGGACCTGAGTCG
	A	TCATCGAACCAGGACTGCAC
DDX43	S	GAGGACTGGTGTTTCCATT
	A	AGCCATTGATACAAGCTCCTC
EPRS	S	AAGGGGAGGTGGTTCGTAAG
	A	GGCTGACCAGGTATGTACTC
ERGIC3	S	AGAACAGCAGCTGGATGTGG
	A	ACCATAGCAGCTCTCACAGC
EXOSC9	S	CTCCTGGGGTGATCTTGAAG
	A	CAACATGGTGAAACCCCAGCT
GPR68	S	GTAGAGCTTGAACCACCTTC
	A	GATGGTATGGTCGATGGTAC
LAMB1	S	ATGACTGCGACCCAGTGACT
	A	GATCTTGGTAGCAGCTCCTG
PBRM1	S	CTGACTGCTGACTTCCAGCT
	A	GTCACTGTGCCCTGATTGTC
RNF126	S	GAGCTTCCGGAAGAGACCAG
	A	GGATCTCGAAGCTGTCATCG
SMCR7L	S	GGTGCCTTCTGTAGACACTC
	A	CACAAAGTCAATGGCCGTGC
TRO	S	GGCTTCAGTGGTGGCCTAAG

	A	CTGCCGAAACTAGCATTGGTG
